# Is sleep duration associated with overweight/obesity in Indigenous Australian adults?

**DOI:** 10.1186/s12889-020-09287-z

**Published:** 2020-08-12

**Authors:** Melissa Deacon-Crouch, Stephen Begg, Timothy Skinner

**Affiliations:** 1 Department of Rural Nursing and Midwifery, La Trobe Rural Health School, La Trobe University, Bendigo, Victoria Australia; 2 Rural Department of Community Health, La Trobe Rural Health School, La Trobe University, Bendigo, Victoria Australia; 3 Department of Psychology, Centre for Health and Society, University of Copenhagen, Copenhagen, Denmark

**Keywords:** Sleep, Indigenous, Obesity, Body mass index, Australian, Adult

## Abstract

**Background:**

Associations between high BMI and sleep duration and chronic illness are recognised. Short sleep is an accepted predictor of high BMI for children, including Indigenous Australian children. Short sleep has also been associated with high BMI in Australian adults, although not specifically in Indigenous Australian adults. This study aims to determine whether the relationship between sleep duration and BMI observed in non-Indigenous adults holds for Indigenous adults.

**Methods:**

Data collected from 5204 non-Indigenous and 646 Indigenous participants aged over 18 years in a nationally representative Australian Health Survey 2011–2013 were analysed. Sleep duration was self-reported as the time between going to bed and time waking up; BMI was derived from measurement and categorised into normal weight (BMI = 18.5–24.9) and overweight/obese (BMI ≥ 25). Logistic regression was performed for the non-Indigenous and Indigenous groups separately to examine the association between sleep duration and BMI in each group.

**Results:**

Proportionally more Indigenous people were classified as overweight/obese than non-Indigenous (χ^2^ = 21.81, *p* < 0.001). Short sleep was reported by similar proportions in both groups (Indigenous 15% vs non-Indigenous 17%) whereas long sleep of > 9 h was reported by proportionally more Indigenous than non-Indigenous people (41% vs 26%). Without accounting for possible confounders, the association between sleep duration and BMI for the Indigenous group was not significant but a possible dose-response relationship was evident, with the odds of overweight/obesity being greatest for those who typically slept < 7 h (OR = 1.77, 95% CI 0.38–3.94) and < 6 h (OR = 1.55, 95%CI = 0.58–4.14). The same model for the non-Indigenous group was significant, with the odds of overweight/obesity being greatest for those who typically slept < 6 h (OR = 1.67, 95%CI 1.25–2.25). The risk of overweight/obesity diminished for both groups with sleep > 7 h. Accounting for a range of socioeconomic and personal confounders attenuated the strength of these relationships marginally.

**Conclusion:**

Adding to reports relating sleep duration and BMI for Australian adults, this study provides evidence for an inverse relationship in non-Indigenous adults and suggests a similar trend for Indigenous adults. This trend was non-significant but is consistent with previous results for Indigenous children.

## Background

High Body Mass Index (BMI) is an accepted risk factor for the development of chronic illnesses including metabolic syndrome and cardiovascular diseases [1, 2]. BMI is calculated as a person’s mass in kilograms divided by their height in metres squared and is categorised by the World Health Organization (WHO) into normal weight (18.5 ≤ BMI < 25), overweight (25 ≤ BMI < 30) and obese (BMI ≥ 30). In the last two decades, Australia has experienced dramatic increases in obesity, while overweight rates have remained relatively constant [3]. From 1995 to 2018 the proportion of obese Australian adults rose from 19 to 31% [4]. Currently, 67% of Australian adults aged 18 years and over are overweight or obese [3]. Indigenous Australians are particularly vulnerable to increased body mass, with nearly three-quarters of adults assessed as overweight or obese (27 and 44%, respectively) [5].

The burden of disease attributable to overweight and obesity in Australia in 2015 was 8.4%, up from 7.0% in 2011, imparting a significant and increasing economic burden [6]. In 2013/2014, the total health expenditure was 38% greater for Indigenous compared with non-Indigenous Australian people [7] reflecting greater health needs of Indigenous people and the higher cost of health services in the regional and rural areas where many Indigenous people live [8].

A number of socioeconomic factors and health behaviours have been identified as contributing to obesity in Australian Indigenous people [9]. However, sleep is one health factor that has not been adequately explored but warrants consideration, given the increasing evidence for an association between sleep duration and BMI in many other populations [10] and because Indigenous people in high income countries like Australia are known to experience poorer sleep than their non-Indigenous counterparts [11–13].

Cardio-metabolic diseases that arise from obesity have been associated with increased cortisol levels and pro-inflammatory processes activation due to the stress response resulting from short sleep duration [14]. High cortisol levels can also stimulate appetite and increase food intake by upregulation of ghrelin and down regulation of leptin, leading to the accumulation of visceral fat [15]. Increased visceral fat leads to insulin resistance and possible obesity due to adverse effects on carbohydrate metabolism [16]. Short sleep duration of less than 7 h has been linked with mortality from all causes and from obesity and cardio-metabolic diseases [17]. A recent review examined the relationship between sleep and cardiometabolic disease risk for global Indigenous populations [13]. The review confirmed a paucity of specific information but concluded that Indigenous populations generally experience suboptimal sleep duration and sleep quality compared with their non-Indigenous counterparts and that poor sleep is likely to negatively impact cardiometabolic health. Regarding Australian Indigenous people, the review reported that Indigenous Australians are 1.8 times more likely to experience obstructive sleep apnoea (OSA) than their non-Indigenous counterpart as a likely result, at least in part, to their higher rates of obesity. The authors also confirmed that, to date, there have been no studies investigating the relationship between sleep and cardio-metabolic risk factors such as obesity, exclusively in Australian Indigenous adults.

Short sleep duration is an accepted predictor of obesity for children, including Indigenous Australian children [18–21]. For adults, the link between sleep duration and BMI seems to depend on age: quasi-linear for young adults; U-shaped in middle age; and less definitive in older age [22]. For Australian adults, short sleep of less than 7 h was associated with obesity in middle aged adults (45–65 years) [23]. However, with age stratification, a U shaped relationship was found for adults aged 55–64 years and no significant relationship for adults over 65 years of age [24]. Similar variation in cross sectional study sleep duration patterns in adults has been previously reported [25]. Information about sleep in Indigenous Australians is lacking [13] and it is not known if sleep duration is associated with obesity in this population.

## Methods

This paper aims to determine whether the relationship between sleep duration and BMI observed in non-Indigenous adults holds for Indigenous adults in a recent, nationally representative sample of the Australian population. It was predicted that short sleep duration would be associated with a higher risk of overweight/obesity in both populations.

### Design and setting

Cross sectional data from the National Nutrition and Physical Activity Survey (NNPAS) [26] and the National Aboriginal and Torres Strait Islander Nutrition and Physical Activity Survey (NATSINPAS) [27] were accessed, analysed and checked through the online Australian Bureau of Statistics (ABS) DataLab. All data are de-identified and protected by the Australian Government Census and Statistical Act 1905. Authorisation to access the Confidentialised Unit Record Files (CURF) from the NNPAS and the NATSINPAS was gained from the ABS in January 2017. Both surveys were components of the Australian Health Survey (AHS) 2011–2013.

More details about the AHS are available in our previous report regarding sleep, BMI and Indigenous children [21]. In brief, the National Aboriginal and Torres Strait Islander Health Survey (NATSIHS) was added to the AHS in 2012–2013. The NATSIHS included a representative sample of around 12,300 Aboriginal and Torres Strait Islander people. The structure and data collection were the same as the AHS including the corresponding NATSINPAS that was conducted in 2900 households, with data collected for one adult and up to one child aged 2–17 years in each selected household. Survey questions in the NATSINPAS were based on those from the NNPAS so the two datasets are comparable and are considered to be nationally representative. The ABS released data for non-remote NATSINPAS participants only. Complete details about the AHS and NATSIHS structure and design, ethics approval and sampling methods and data collection are described elsewhere [28, 29].

### Procedures

Trained ABS interviewers conducted face-to-face interviews with the selected adult member of all households.

### Measures

#### Sleep duration

reported as the time between going to bed and time waking up (hours and minutes). ‘Time went to bed’ was defined as time the light was turned off with the intention of going to sleep, as it can be difficult to ascertain the time when sleep commences. ‘Time woke up’ was defined as ‘the last time waking up’. A typical night’s sleep was a self-reported measure by respondents. Sleep duration was stratified into 5 categories: < 6 h, 6 h (≥6 to < 7 h), 7 h (≥7 to < 8 h), 8 h (≥8 to < 9 h) and ≥ 9 h [30].

#### BMI

Height and weight were objectively measured by the interviewers. Calculated BMI was categorised using World Health Organisation (WHO) guidelines: normal (18.5–24.9), overweight (25–29.99) and obese (greater than or equal to 30) [31]. The overweight and obese groups were combined in this analysis such that BMI was dichotomised into overweight/obese (high BMI) versus normal weight (normal BMI).

#### Index of relative socio-economic disadvantage – 2011 (SEIFA)

is a general socio-economic index that summarises a range of information about the economic and social conditions of people and households within an area. The index includes measures of relative disadvantage including low income, low educational attainment, high unemployment and jobs in relatively unskilled occupations. The index relates to the area in which the survey respondent lived and is not necessarily indicative of their individual socio-economic status.

#### Equivalised income of household (deciles)

Standardised estimates of income using the modified OECD equivalence scale (1994) reflect the households’ relative well-being allowing for differences in household types, compositions and requirements relative to income.

#### Australian statistical geography standard (ASGS) (2011)

was used by the ABS to generate two broad geographical categories; non-remote (including major cities and inner regional Australia) and remote. However, the ABS only released the non-remote data for the Indigenous sample so we excluded the remote participants for the non-Indigenous sample to ensure comparability.

#### Fruit and vegetable consumption

was used as a measure of dietary behaviour which was categorised by the ABS as either having met the Australian Dietary Guidelines [32] or not.

#### Physical activity

was categorised by the ABS as having met 150 min of physical activity per week as recommended by the *Australian Physical Activity and Sedentary Behaviour Guidelines for Adults* [33] or not.

### Statistical analyses

The variables of interest that were common to both the NNPAS and the NATSINPAS databases were identified (Table 1). Data were cleaned by excluding implausible values, missing data and outliers. A final merged dataset of NNPAS and NATSINPAS was restricted to adults aged 18 years and older who had identified the previous night’s sleep duration as ‘typical’. Typical night’s sleep reports were considered to reflect the approximate pattern and/or number of hours of sleep that occurred on the same night of the week over time. Demographic, categorical data for the two groups were compared using χ^2^ tests. Logistic regression analyses were performed for the non-Indigenous and Indigenous groups separately to examine the association between sleep duration and BMI in both groups. Two models were tested for each group. Model 1: the non-adjusted model examined the relationship between sleep duration and BMI. Model 2: the adjusted model examined the relationship between sleep duration and BMI controlling for the covariates described in Table 1. Sleep duration of 7 h was used as the reference category because it has been reported as having the lowest mortality risk [30]. To reduce the risk of Type 1 error due to multiple testing, the OR were tested against a *p* value of 0.025 (two-tailed). All analyses were completed through the ABS *DataLab* using STATA 13 [34].

## Results

There were 646 people in the Indigenous group and 5204 people in the non-Indigenous group.

The Indigenous group was significantly younger than the non-Indigenous group: t (5848) =9.78, *p* < .001 (42 ± 15.5 vs 49 ± 16.2 years of age, respectively).

Table 1 describes the characteristics of the non-Indigenous and Indigenous groups including gender, socioeconomic factors, typical daily sleep duration, physical activity, dietary factors, smoking status and BMI. Results show that short sleep (< 7 h/night) was experienced by similar proportions of participants in both groups (17% non-Indigenous, 15.5% of Indigenous participants). A greater proportion of Indigenous participants reported long sleep compared with non-Indigenous people (> 9 h/night: 40.7% vs 25.9%, χ^2^(4) =67.59, *p* < 0.001 respectively). Compared with the non-Indigenous group, the Indigenous group had a greater proportion of female participants; were more likely to live in households with 3 or more children under 17 years of age; and were over represented in the most deprived quintile (50.6%, quintile 1) and underrepresented in the least deprived quintile (3.3%, quintile 1). Significantly more Indigenous people were current smokers than non-Indigenous participants (42% vs 16.2%, χ^2^(3) = 257.45, *p* < 0.001) and significantly fewer met the recommended Australian physical activity guidelines each week compared with non-Indigenous participants (55.6% vs 49%, χ^2^(1) =9.93, *p* < 0.005). A significantly greater proportion of Indigenous people were classified as overweight/obese than non-Indigenous people (73.8% vs 64.6%, χ^2^(1) = 21.81, *p* < 0.001).

**Table 1:** Demographic information for Indigenous and Non-Indigenous adults who reported sleep duration as ‘typical’

Category	Sub-Category	non-Indigenous ***N*** = 5204	Indigenous ***N*** = 646	***χ***^***2***^	df	p
		N (%)	N (%)			
**Gender**	Males	2504 (48.12)	270 (41.80)	1	9.21	< .005
	Female	2700 (51.88)	376 (58.20)			
**Index of Relative Socio-economic Disadvantage – 2011 (SEIFA)**	Quintile 1(lowest)	996 (19.14)	327 (50.62)	4	395.29	< .001
	Quintile 2	1106 (21.25)	160 (24.77)			
	Quintile 3	1022 (19.64)	74 (11.46)			
	Quintile 4	1040 (19.98)	64 (9.91)			
	Quintile 5highest)	1040 (19.98)	21 (3.25)			
**Labour Force Status**	Employed	3263 (62.70)	283 (43.81)	2	138.73	< .001
	Unemployed	120 (2.31)	57 (8.82)			
	Not in the labour force	1821 (34.99)	306 (47.37)			
**Equivalised income of household: quintiles**	Quintile 1 (lowest income)	1088 (20.91)	278 (43.03)	4	222.98	< .001
	Quintile 2	1002 (19.25)	162 (25.08)			
	Quintile 3	918 (17.64)	89 (13.78)			
	Quintile 4	1096 (21.06)	73 (11.30)			
	Quintile 5 (highest income)	1100 (21.14)	44 (6.81)			
**Household type**	person living alone	1442 (27.71)	145 (22.45)	5	186.04	< .001
	Couple only	1537 (29.53)	116 (17.96)			
	Couple family with children	1447 (27.81)	169 (26.16)			
	One parent family with children	421 (8.09)	120 (18.58)			
	Unrelated persons aged ≥15 yrs	124 (2.38)	10 (1.55)			
	All other households	233 (4.48)	86 (13.31)			
**Number of adults in household**	One	1703 (32.72)	234 (36.22)	3	5.24	>.05
	Two	2854 (54.84)	324 (50.15)			
	Three	466 (8.95)	65 10.06)			
	Four or more	181 (3.48)	23 (3.56)			
**Number of children in household (0 – 17 years)**	None	3642 (69.98)	331 (51.24)	4	143.5	< .001
	One	619 (11.89)	103 (15.94)			
	Two	631 (12.13)	109 (16.87)			
	Three	226 (4.34)	59 (9.13)			
	Four or more	86 (1.65)	44 (6.81)			
**Sleep duration categories**	< 6 h	285 (5.48)	35 (5.42)	4	67.59	< .001
	6 - < 7 h	594 (11.48)	65 (10.06)			
	7 - < 8 h	1337 (25.69)	111 (17.18)			
	8- < 9 h	1638 (31.48)	172 (26.63)			
	≥9 h	1350 (25.94)	263 (40.71)			
**Body Mass Index (BMI)Categories**	Normal rangeBMI 18 to < 25	1843 (35.42)	169 (26.16)	1	21.81	< .001
	Overweight/ObeseBMI ≥ 25	3361 (64.58)	477 (73.84)			
**Vegetable** **&** **fruit consumption**	Met recommended guidelines	286 (5.50)	32 (4.95)	1	0.33	>.05
	Did not meet recommended guidelines	4918 (94.50)	614 (95.05)			
**Physical activity last week at least 150 min**	Met recommended guidelines	2654 (51.00)	287 (44.43)	1	9.93	< .005
	Did not meet recommended guidelines	2550 (49.00)	359 (55.57)			
**Smoking status**	current - daily	845 (16.24)	271 (41.95)	3	257.45	< .001
	current - weekly or less	92 1.77)	13 (2.01)			
	Ex-smoker	1773 (34.07)	185 (28.64)			
	Never smoked	2494 (47.92)	177 (27.40)			

Unadjusted and adjusted odds ratios for the Indigenous and non-Indigenous groups are shown in Table 2. In the unadjusted models, sleep duration alone accounted for very little of the overall variance in the odds of overweight/obesity in either group (non-Indigenous pseudo R^2^ = 0.005, *p* < 0.001 and Indigenous pseudo R^2^ = 0.012, *p* = 0.07, respectively). The unadjusted model for the Indigenous group was not significant but nevertheless a possible dose-response relationship was evident, with the odds of obesity being greatest for Indigenous people who typically slept less than 7 h (OR = 1.77, 95% CI 0.79–3.94) and < 6 h (OR = 1.55, 95%CI = 0.58–4.14). The unadjusted model for the non-Indigenous group was significant, with the odds of overweight/obesity being greatest for those who typically slept less than 6 h (OR = 1.67, 95%CI 1.25–2.25). Longer than normal sleep times seem to be associated with lower odds of overweight/obesity for both Indigenous and non-Indigenous people; however, the risk reduction was only statistically significant for non-Indigenous people who typically slept for more than 9 h (OR = 0.81, 95%CI 0.69–0.95).

**Table 2:** Unadjusted and adjusted odds ratios for Indigenous and non-Indigenous groups

	Indigenous		non-Indigenous	
	N = 646		N = 5204	
	OR	95% CI	OR	95% CI
**Model 1**	**p **= 0.075**		**p < 0.001	
**Sleep duration (hrs)**				
< 6	1.55	(0.58–4.14)	1.67*	(1.25–2.25)
< 7	1.77	(0.79–3.94)	1.22	(0.99–1.50)
< 8	**Ref**		**Ref**	
< 9	0.88	(0.51–1.53)	0.9	(0.77–1.04)
9+	0.74	(0.44–1.22)	0.81*	(0.69–0.95)
**Model 2**	**p < 0.001		**p < 0.001	
**Sleep duration (hrs)**				
< 6	1.51	(0.53–4.30)	1.52*	(1.12–2.06)
< 7	1.66	(0.67–4.07)	1.15	(0.93–1.43)
< 8	**ref**		Ref	
< 9	0.71	(0.38–1.31)	0.92	(0.78–1.07)
9+	0.77	(0.43–1.37)	0.88	(0.74–1.03)
**Age**	1.04*	(1.03–1.06)	1.03*	(1.02–1.03)
**Female**	0.64	(0.41–1.01)	0.56*	(0.49–0.63)
**Employment Status**				
Employed	**Ref**		**Ref**	
Unemployed	0.81	(0.38–1.73)	0.96	(0.64–1.42)
Not in Labour force	0.99	(0.58–1.71)	0.8*	(0.67–0.95)
**Socioeconomic status of locality**				
Quintile 1 (lowest)	**Ref**		**Ref**	
Quintile 2	0.89	(0.55–1.44)	0.92	(0.76–1.11)
Quintile 3	1.84	(0.91–3.71)	0.71*	(0.59–0.87)
Quintile 4	0.56	(0.29–1.10)	0.66*	(0.54–0.81)
Quintile 5 (highest	0.86	(0.24–3.12)	0.6*	(0.49–0.74)
**Household type**				
Person living alone	**Ref**		**Ref**	
Couple only	4.64*	(1.44–14.98)	1.47	(0.89–2.42)
Couple family with children	8.16*	(1.59–41.86)	1.62	(0.81–3.22)
One parent family with children	3.48*	(1.16–10.47)	1.27	(0.84–1.93)
Unrelated persons aged 15+ only	0.96	(0.15–6.27)	1.14	(0.60–2.15)
All other households	5.46*	(1.14–26.17)	1.65	(0.81–3.36)
**Equivalised income of household**				
Quintile 1 (lowest)	**Ref**		**Ref**	
Quintile 2	1.48	(0.85–2.56)	1.2	(0.99–1.46)
Quintile 3	1.3	(0.62–2.71)	1.1	(0.88–1.36)
Quintile 4	1.05	(0.48–2.29)	1.31*	(1.05–1.64)
Quintile 5 (highest	1.75	(0.56–5.15)	1.34*	(1.07–1.69)
**Number of adults in household**				
One	**Ref**		**Ref**	
Two	0.49	(0.19–1.24)	0.71	(0.44–1.14)
Three	0.5	(0.15–1.62)	0.65	(0.35–1.17)
Four or more	1.05	(0.21–5.16)	0.59	(0.31–1.16)
**Number of children 0–17 yrs. in household**				
None	**Ref**		**Ref**	
One	0.59	(0.24–1.47)	1	(0.73–1.39)
Two	0.48	(0.18–1.27)	0.96	(0.68–1.35)
Three	0.69	(0.24–2.00)	1.28	(0.84–1.94)
Four or more	0.46	(0.15–1.40)	0.92	(0.54–1.58)
**Fruit and vegetable intake**				
Met recommended dietary guidelines	**Ref**		**Ref**	
Did not meet recommended dietary guidelines	2.00	(0.85–4.76)	1.05	(0.81–1.36)
**Physical activity**				
Met recommended guidelines	**Ref**		**Ref**	
Did not meet recommended guidelines	1.37	(0.92–2.05)	1.2*	(1.06–1.35)
**Smoking Status**				
Current daily	**Ref**		**Ref**	
Current weekly or but not daily	1.96	(0.54–7.20)	0.9	(0.57–1.41)
Ex-smoker	3.16*	(1.84–5.43)	1.44*	(1.20–1.73)
Never smoked	1.75*	(1.07–2.86)	1.08	(0.91–1.28)

* indicates significant result **indicates the overall *p* value for the model

**Fig. 1:**
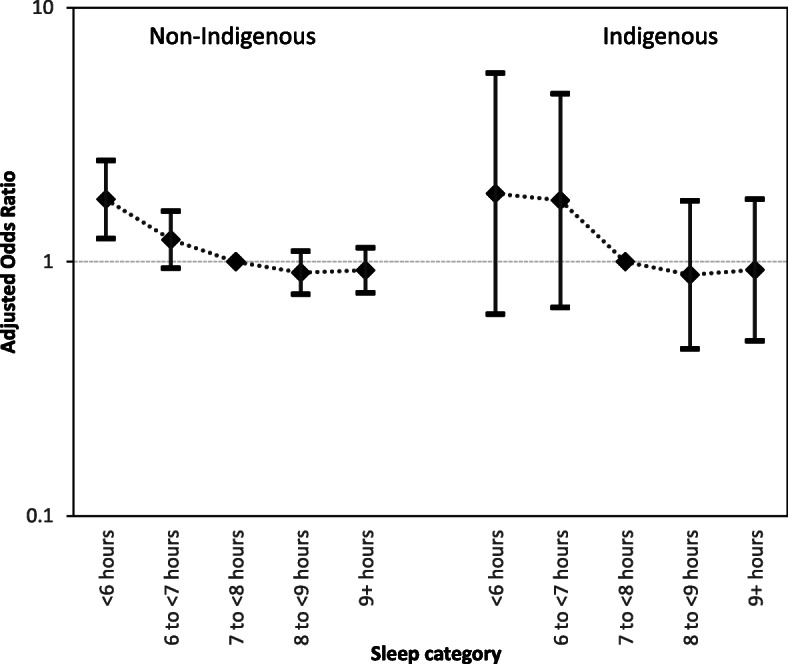
Adjusted Odds Ratios for overweight/obesity related to sleep duration for non-Indigenous and Indigenous people

The inclusion of covariates marginally increased the overall explanatory power of the models (non-Indigenous pseudo R^2^ = 0.06, *p* < 0.001 and Indigenous pseudo R^2^ = 0.15, p < 0.001, respectively). For the Indigenous group, the same trends observed in the unadjusted model were evident in the adjusted model, but again the results were not statistically significant. For the non-Indigenous group, the odds of overweight/obesity were statistically significant for those who slept less than 6 h (OR = 1.52, 95%CI = 1.12–2.06). Like the unadjusted model, the adjusted model suggested that longer than 7 h sleep lowered the odds of overweight /obesity for the Indigenous and non-Indigenous groups alike (see Fig. 1). For both groups, there seemed to be an inverse relationship between sleep duration and the odds of overweight /obesity.

For Indigenous participants, the two covariates in the adjusted model that were most strongly associated with overweight/obese were household type and smoking status. Compared with living alone, there was significantly greater odds of being overweight/obese if the person lived as part of a couple without children (OR = 4.64, 95%CI = 1.44–14.98); with a partner and children (OR = 8.16, 95%CI = 1.59–41.86); as a single parent family (OR = 3.48, 95%CI = 1.16–10.47); and in any other type of household other than with unrelated people (OR = 5.46, 95%CI = 1.14–26.17). Indigenous people who were ex-smokers (OR = 3.16, 95%CI = 1.84–5.43) or who had never smoked (OR = 1.75, 95%CI = 1.07–2.86) were at significantly greater odds of being overweight/obese compared with daily smokers.

Socioeconomic status of home locality and household income were not significantly associated overweight/obesity, although the odds seemed lowest for those who lived in the higher socioeconomic localities.

For non-Indigenous participants, the odds of overweight /obesity were significantly lower in females than males (OR = 0.56, 95%CI = 0.49–0.63). Similarly, people not participating in the labour force had a significantly lower odds of overweight/obesity compared with those in employment (OR = 0.80, 95%CI = 0.67–0.95). The odds of overweight/obesity was greatest for those living in the lowest socioeconomic localities, with a significantly lower odds for those living in the mid to higher socioeconomic status localities (quintile 3 (midrange): OR = 0.71, 95%CI = 0.59–0.87; quintile 4: OR = 0.66, 95%CI = 0.54–0.81; quintile 5(highest): OR = 0.60, 95%CI = 0.49–0.74). However, the opposite is observed for actual household income where participants living in the household with the highest incomes had the greatest odds of being overweight/obese (quintile 4: OR = 1.31, 95%CI = 1.05–1.64 and quintile 5 (highest): OR = 1.34, 95%CI = 1.07–1.69). The risk of overweight/obesity was significantly greater for non-Indigenous people who did not meet the weekly physical activity guidelines (OR = 1.2, 95%CI =1.06–1.35) and for ex-smokers (OR = 1.44, 95%CI = 1.20–1.73).

Increased age was significantly associated with overweight/obese in both groups (Indigenous OR = 1.05, 95%CI 1.03–1.07); non-Indigenous OR = 1.03, 95%CI =1.03–1.04). Fruit and vegetable intake did not significantly impact on the risk of overweight/obesity for either group, however Indigenous participants had twice the risk of overweight/obesity associated with inadequate dietary intake of fruit and vegetables (OR = 2.00, 95% CI = 0.85–4.76) compared with Indigenous people who met the dietary guidelines. Indigenous participants who did not meet the recommended 150 min of physical activity per week were also at higher risk of overweigh/obesity compared with those who did meet the guidelines, although again the results were not significant for this group (OR = 1.37, 95%CI = 0.92–2.05).

## Discussion

Through its association with chronic cardiometabolic diseases, excess BMI has been established as a leading contributor to the disease burden in Australia [3]. This is particularly so for Indigenous people who are at higher risk of obesity based on BMI measurements [9] as demonstrated by this study, where significantly more Indigenous people were classified as overweight/obese than non-Indigenous people. For the past two decades, reports have also established an association between BMI and sleep duration, indicating that sleep durations ≤7 h and ≥ 9 h, are not ideal for health and may influence BMI [35, 36].

Previous Australian studies have shown that for adults aged 45–65 years, short sleep (< 7 h) was associated with obesity in both males and females while long sleep (≥9 h) was associated with obesity in males only [30]. Further stratification showed that both short and long sleep were associated with obesity (U-shaped curve) for those aged 55–65 years [23]; however, there was no association between sleep duration and obesity for those over 65 years [24].

Due to the comparatively small number of Indigenous adults in our study, we did not stratify participants by age. Contrasting with previous reports, our results indicate that Indigenous adults are more likely to experience long sleep duration compared with non-Indigenous adults [12, 36]. Furthermore, rather than a previously described U-shaped curve [23], we found evidence of an inverse relationship between sleep duration and BMI, with longer sleep duration appearing to reduce the odds of developing overweight/obesity for both Indigenous and non-Indigenous participants.

In our analysis, similar to previous meta-analysis guidelines, we considered short sleep duration to be < 7 h, optimal sleep duration to be 7–9 h with 7–8 h used as the normal reference range and long sleep to be ≥9 h [17]. Exploring the relationship between sleep duration and BMI in the unadjusted model, without consideration of other possible confounders, both Indigenous and non-Indigenous people who slept less than 7 h per night (i.e. short sleepers) were more likely to be overweight/obese compared with their counterparts who slept a normal 7–8 h (i.e. the reference sleeping category) on a typical night. Even though both the Indigenous and non-Indigenous groups reflected similar trends in this respect, only the results for the non-Indigenous group were statistically significant. The absence of significant association in the Indigenous group is most likely a power issue, with the Indigenous group being approximately eight times smaller than the non-Indigenous group.

In the adjusted model, the combination of covariates accounted for a greater proportion of the variance in BMI for Indigenous people compared with the non-Indigenous people. Like the unadjusted model, the relationship between sleep duration and BMI in the adjusted model showed that both Indigenous and non-Indigenous people who slept less than 7 h per night were more likely to be overweight/obese compared with those who slept a normal 7–8 h on a typical night. Preliminary reports from a 6 year longitudinal study indicated that short sleepers gained more abdominal fat than people who slept for 7 or more hours [15]. This is important because fat deposition patterns tend to favour the abdomen in the Australian Indigenous population, regardless of overall weight [37]. Hence, BMI-based measurements are likely to underestimate this risk in Indigenous Australians and the prevalence of actual overweight and obesity may be much greater than reported.

Consistent with the findings of a meta-analysis [10], our study showed that more than 9 h of sleep did not pose a greater likelihood of overweight/obesity than sleeping 7–8 h. The reasons for weight gain with longer sleep are thought to be different from that of short sleep duration [38]. Furthermore, it is difficult to quantify the effect of long sleep on BMI because often adults are already overweight/obese when recruited to studies [39] and their long sleep may be attributable to other comorbidities such as depression, cancer and the effects of medications [40]. In light of this, and given that in this study, a larger proportion of Indigenous people compared with non-Indigenous people reported long sleep duration, it is important to note that other Australian reports have shown that Indigenous adults consistently self-report mental health concerns at higher rates than other members of the Australian community [41].

There is a paucity of information about the relationship between mental health and sleep for Australian Indigenous people. Unfortunately, such an analysis was not possible in this study as mental health data was not included in the survey we accessed. Notably however, one American study of middle-aged women with ongoing depressive symptoms found the women to be almost three times more likely to have long sleep of 9 or more hours per night, compared with those who did not experience depression [40]. Another study of adults aged over 57 years from the Netherlands also showed that people who experienced long sleep (≥ 9 h per night) were significantly more likely to have a depressive disorder than people who slept 7 to < 8 h per night [42]. Exploration of such associations in the Australian Indigenous community are for future research.

Considering other covariates associated with obesity, increasing age was significant for both Indigenous and non-Indigenous people. This may reflect that as people age, they are more likely to suffer chronic comorbidities and be less physically active [20, 22]. Gender differences have been previously reported whereby Indigenous Australian women have been found to be significantly more likely to be obese compared with Indigenous males [9]. However, in this study, gender differences were apparent only in the non-Indigenous group where males were significantly more likely to be overweight/obese than females. Greater activity levels in Australian females compared with males may explain this finding [36]. Overall, participation in recommended levels of physical activity was lower for both Indigenous and non-Indigenous participants in this study than reported for another Australian study [36]. However, there seemed to be an increased risk, albeit non-significant, of overweight/obesity for Indigenous participants who did not meet the guidelines compared with those who did, which may be due to short sleep leading to increased daytime fatigue and lower levels of physical activity [43], long sleep leading to less time for physical activity or, if the long sleep is related to other comorbidities, long sleepers having less capability for physical activities [44].

Family life generally seemed to be associated with overweight /obese for both groups, however our results were only significant for the Indigenous group. Indigenous people living with a partner or children under 18 were significantly more likely to be overweight or obese compared to people living alone. Similarly, in the non-Indigenous group, those living in families with children under 18 were at greatest risk of being overweight or obese. A recent Australian study showed that compared with singles, couples were significantly less likely to be within a normal weight range [45], while another associated lower levels of physical activity in the time of transition to parenthood as a main contributor to increased obesity of parents [46].

Our findings of the greater risk of overweight/obesity in Australian males and for those who are not living alone are consistent with other studies of males living in Tehran and the Gulf States, which showed that overweight and obesity were significantly more prevalent in those who were married [47] or living with a partner and other family members compared with those who lived alone [48]. Socioeconomic status of the household locality did not significantly increase the odds of overweight/obesity for Indigenous people, however the trend appeared to be that people who resided in higher socioeconomic areas were less likely to be overweight/obese than those who lived in the most deprived areas. The significantly lower SEIFA ranking and household income may reflect a greater number of Indigenous women in the sample caring for greater numbers of children.

Results for the non-Indigenous people reflected this trend and were statistically significant. Non-Indigenous people living in the 3 highest quintile areas were significantly less likely to be overweight/obese compared with those living in the lowest quintile areas. These findings are consistent with recent reports that show the proportion of Australian adults aged at least 18 years who were overweight or obese increased with relative disadvantage [9].

The results for nutritional intake were not statistically significant but suggested Indigenous participants with inadequate dietary intake of fruit and vegetables had double the odds of overweight/obesity associated compared with those who met the dietary guidelines when other covariates were accounted for. This finding is consistent with a recent meta-analysis including of Australian studies, that reported an inverse relationship between fruit and vegetable consumption and risk of increased adiposity [49].

Finally, compared with current smokers, all other Indigenous participants were at increased odds of overweight/obesity, with the odds being greatest amongst ex-smokers. Weight gain following smoking cessation is well documented, and there are concerns that the benefits of tobacco cessation maybe diminished by the onset of overweight/obesity and the associated comorbidities [50].

## Limitations

Several limitations must be considered in relation to the findings presented here. First, the study presents prevalence data and the use of cross-sectional data precludes the determination of causality and possible direction of causation. However, it is probable that the association between short sleep duration and greater than normal BMI is bidirectional. As already mentioned, overweight/obesity may result from prolonged short sleep duration or it may be that overweight/obesity causes short sleep due to concomitant comorbidities and pain. Obstructive sleep apnoea (OSA) is one such comorbidity that has been associated with overweight/obesity [51] and sleep disturbance [52]. OSA could not be considered in this study because it was not part of the survey dataset we accessed but a recent review concluded that it is more prevalent and more severe in global Indigenous populations than non-Indigenous populations [12]. Two reports have since added to the Australian Indigenous context. A retrospective analysis of diagnostic sleep test data from the Northern Territory found that severe sleep apnoea was associated with male gender, increasing age, higher body weight, and Indigenous status [53]. Similar data from Central Australia and far north Queensland reported that compared with non-Indigenous people, Indigenous people diagnosed with OSA were more likely to be female, live in remote communities and be obese [54]. This is an area for further research given the prevalence of obesity in Indigenous communities and the associated risk of OSA with adverse health outcomes and increased all-cause mortality [53].

Second, self-reported sleep data, although widely used, is likely to systematically overestimate sleep duration. It is very difficult, for example, for a person to know when sleep actually occurs without objective measurement. If sleep time is calculated from the time the person intended to go to sleep and it took longer than anticipated to fall asleep, this would lead to an overestimation of sleep duration. However, it is unlikely that such biases affected the Indigenous and the non-Indigenous groups in this analysis differentially. Although actigraphy and polysomnography are considered better objective measurements of sleep duration [25], there was no opportunity to such measures in this study.

Third, BMI was used in this study because it is the most commonly used measure of overweight / obesity [55]. However, World Health Organisation recommended BMI ranges may be inappropriate for Indigenous Australians [56] and may, in fact, underestimate overweight / obesity in this population. Waist measurements of ≥94 cm for men or ≥ 80 cm for women are recognised as better measures of body fat and are indicative of increased risk of developing chronic disease such as heart disease and Type 2 diabetes (WHO, 2000). High BMI may be indicative of a much greater risk and link with metabolic syndrome and chronic illness for Indigenous Australians than previously thought. It is reassuring, therefore, that in this sample at least, there was a strong correlation between the two measures (r = 0.9, *n* = 16,262, *p* < 0.01).

## Conclusion

The results of this study differ from the previously reported 45 up Australian study [23] in that in there appeared to be an inverse relationship between sleep duration and BMI for non-Indigenous Australians and an inverse trend between sleep duration and BMI for Indigenous Australians, rather than the previously reported U-shaped relationship [24]. In this study, BMI appears to be partially explained by current sleep duration in non-Indigenous adults, but this pattern is less well defined in Indigenous adults. Sleep duration did not appear to be strongly associated with BMI for either non-Indigenous or Indigenous participants. The adjusted model indicates that other factors are more strongly associated with overweight/obesity than sleep. Further research is needed to determine whether the null findings with sleep duration and BMI among the Indigenous sample can be explained by the interaction between social and personal determinants of health and Indigenous status, and whether sleep duration plays a mediating or moderating role in the predisposition of Indigenous Australians towards overweight/obesity.

Interestingly, a recent study of Australian Indigenous children found that sleep duration is inversely associated with overweight /obesity [57], while a cascade model of paediatric obesity identified that despite a small effect size, short sleep duration is a key factor in the development of obesity in school-aged children [58] . Cascade models have the basic tenet that no single risk or protective factor has a large effect but its early and cumulative influence snowballs to a greater effect over time. Such models may be useful to explain the cumulative consequences and downstream effects of the complex multifactorial risk and protective factors associated with adult obesity. Using such a model, it may be that the influence of sleep on future adult BMI is most potent in childhood.

Another piece of the puzzle that needs to be considered is that a previous longitudinal study of non-Indigenous adults found a positive effect by optimising sleep duration over a six-year period. People with a baseline short sleep duration (≤ 6 h per day) who increased their sleep to 7–8 h over the 6-year period experienced significantly lower adiposity gain than those who maintained their short sleep habit but no significant difference in adiposity gain compared with the control group who maintained their baseline 7–8-h sleep duration [15].

In view of the cascade model of paediatric obesity and the positive effects of sleep optimisation found in non-Indigenous people, further work is needed, ideally in a prospective longitudinal study, where sleep can be measured over childhood and adulthood to gain a clearer picture of the role of sleep duration in the health and wellbeing of Australian Indigenous people.

## Data Availability

The datasets generated analysed during the current study are available on successful application from the Australian Bureau of Statistics DataLab, NNPAS**:** https://www.abs.gov.au/AUSSTATS/abs@.nsf/Lookup/4324.0.55.002Main+Features12011-12 NATSINPAS**:** https://www.abs.gov.au/ausstats/abs@.nsf/Lookup/4715.0.30.002main+features12012-13

## Abbreviations

ABS: Australian Bureau of Statistics
AHS: Australian Health Survey
ASGS: Australian Statistical Geography Standard
BMI: Body Mass Index
CURF: Confidentialised Unit Record File
NATSINPAS: National Aboriginal and Torres Strait Islander Nutrition and Physical Activity Survey
NNPAS: National Nutrition and Physical Activity Survey
SEIFA: Index of Relative Socio-economic Disadvantage
WHO: World Health Organisation
